# Giant omphalocele: A novel approach for primary repair in the neonatal period using botulinum toxin

**DOI:** 10.1590/0100-6991e-20233582-en

**Published:** 2023-11-10

**Authors:** Marcelo Costamilan Rombaldi, Caroline Gargioni Barreto, Letícia Feldens, Felipe Holanda, Eliziane Emy Takamatu, Luciano Schopf, Carlos Alberto Hoff Peterson, Eduardo Corrêa Costa, Leandro Totti Cavazzola, Paola Isolan, José Carlos Fraga

**Affiliations:** 1 - Universidade Federal do Rio Grande do Sul, Programa de Pós-Graduação em Medicina: Ciências Cirúrgicas - Porto Alegre - RS - Brasil; 2 - Hospital de Clínicas de Porto Alegre, Departamento de Cirurgia Pediátrica - Porto Alegre - RS - Brasil; 3 - Hospital de Clínicas de Porto Alegre, Departamento de Cirurgia Geral - Porto Alegre - RS - Brasil; 4 - Universidade Federal do Rio Grande do Sul, Departamento de Cirurgia - Porto Alegre - RS - Brasil

**Keywords:** Hernia, Umbilical, Botulinum Toxins, Infant, Newborn, Abdominal Wall, Abdominal Wound Closure Techniques, Hérnia Umbilical, Toxinas Botulínicas, Recém-Nascido, Parede Abdominal, Técnicas de Fechamento de Ferimentos Abdominais

## Abstract

**Introduction:**

Giant omphalocele (GO) is a complex condition for which many surgical treatments have been developed; however, no consensus on its treatment has been reached. The benefits and efficacy of botulinum toxin A (BTA) in the repair of large abdominal wall defects in adults has been proven, and its reported use in children has recently grown. The goal of this study is to describe a novel technique for primary repair of GO using BTA during the neonatal period and report our initial experience.

**Methods:**

patients were followed from August 2020 to July 2022. BTA was applied to the lateral abdominal wall in the first days of life followed by surgical repair of the abdominal defect.

**Results:**

while awaiting surgery, patients had minimal manipulation, without requiring mechanical ventilation, were on full enteral feeding, and in contact with their parents. The midline was approximated without tension and without the need for additional techniques or the use of a prosthesis. Patients were discharged with repaired defects.

**Conclusion:**

this approach represents a middle ground between staged and the nonoperative delayed repairs. It does not require aggressive interventions early in life, allowing maintenance of mother-child bonding and discharge of the patient with a repaired defect without the need for additional techniques or the use of a prosthesis. We believe that this technique should be considered as a new possible asset when managing this complex condition.

## RESUMO

**Introdução:**

onfalocele gigante (OG) é uma condição complexa para a qual muitas alternativas terapêuticas foram desenvolvidas; no entanto, não há consenso sobre qual o melhor tratamento. Os benefícios e eficácia da toxina botulínica A (TBA) no reparo de grandes defeitos da parede abdominal em adultos foram comprovados, e o relato de uso em crianças cresceu recentemente. O objetivo deste estudo é descrever uma nova técnica para reparo primário de OG utilizando TBA durante o período neonatal e relatar nossa experiência inicial.

**Métodos:**

os pacientes foram acompanhados de agosto de 2020 a julho de 2022. A TBA foi aplicada na parede abdominal lateral nos primeiros dias de vida, seguida de correção cirúrgica do defeito abdominal.

**Resultados:**

enquanto aguardavam a cirurgia, os pacientes tiveram mínima manipulação, sem ser exigida ventilação mecânica, permaneceram em alimentação enteral plena e mantiveram contato com os pais. A linha média foi aproximada sem tensão e sem necessidade de técnicas adicionais ou uso de prótese. Os pacientes receberam alta com o defeito reparado.

**Conclusão:**

essa abordagem representa um meio-termo entre o reparo estagiado e o tratamento não-operatório. Não requer intervenções agressivas no início da vida, permitindo a manutenção do vínculo materno-infantil e a alta do paciente com defeito reparado sem a necessidade de uso de técnicas adicionais ou prótese. Acreditamos que esta técnica deva ser considerada como um novo possível recurso no manejo desta complexa condição.

## INTRODUCTION

Omphalocele is a congenital defect of the abdominal wall that occurs due to the persistence of physiological herniation of the midgut^1^. The size of the defect and the content of the hernial sac are variable, ranging from small defects containing hollow viscera to larger defects containing solid organs.

Its prevalence is estimated between 1 to 3.8/10,000 live births^2^, and up to 80% of patients have associated malformations, most commonly cardiovascular^3^. The associated malformations make the treatment of omphalocele a challenge.

Giant omphalocele (GO) is considered a more complex variation of the spectrum and is characterized by the association of a large wall defect (>5cm) and a voluminous hernia sac (usually with the presence of the liver), with an important visceroabdominal disproportion^4^. Patients with GO often have pulmonary hypoplasia, which can significantly increase neonatal morbidity and mortality, especially when associated with other anomalies^5^ ^,^ ^6^.

There are two main strategies for managing GO: staged surgical closure and non-operative delayed closure. However, there is no consensus and there is evidence to support both approaches^7^ ^,^ ^8^ being the choice guided by clinical presentation, patient comorbidities and surgeon preference.

Recently, the use of botulinum toxin A (BTA) in the repair of abdominal hernias with loss of domain in the adult population has become more frequent, and its use has already been shown to facilitate the closure of the abdominal wall^9^. In the pediatric population, our group described the first cases in the literature of the use of BTA in children with ventral hernias secondary to omphalocele, with safe and favorable results^4^ ^,^ ^10^. Although some authors have also described the use of BTA, in association with other techniques and in older children^11^ ^,^ ^12^, this was the first detailed description and report of the isolated use of BTA in neonates for primary correction of GO.

The aim of this study is to describe the technique and methodology for applying BTA in patients with GO during the neonatal period and to report the initial experience, favorably resulting in primary closure of the abdominal wall without the use of additional techniques or prostheses.

## METHODS

Written consent was obtained from the patients’ legal guardians. This study was approved by the Ethics in Research Committee on October 20, 2022 (CAAE 62606922.7.0000.5327, opinion 5711519).

### BTA application technique Injection technique

The application of BTA was performed in the Neonatal Intensive Care Unit (NICU) under sedation, ultrasound guidance, and sterile conditions. We chose a neonatal spinal needle, burt a regular 25mm x 0.7mm (22G 1/4) needle could also be used.

**Figure 1:**
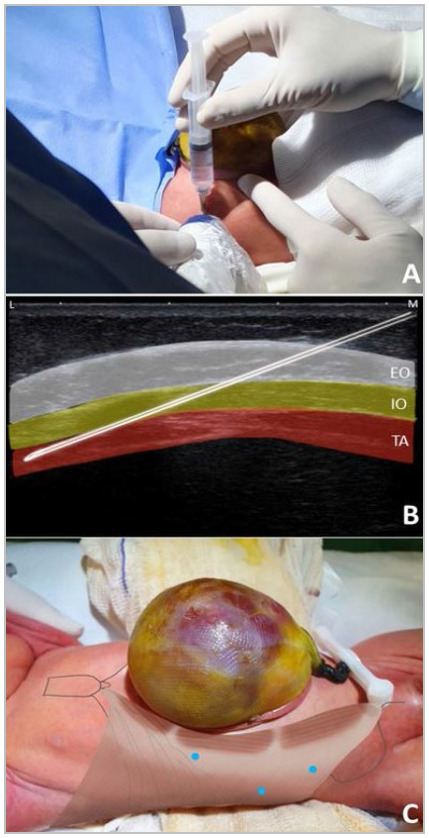
BTA application technique on the lateral abdominal wall of a newborn with GO. (A) The transducer is placed transversely and the needle in the medial-lateral direction. (B) Ultrasound image with the three differentiated muscle layers and correct positioning of the needle; white: external oblique muscle, yellow: internal oblique muscle, red: transversus abdominis muscle. (C) Blue dots representing injection sites.

The ultrasound transducer was transversely positioned on the lateral abdominal wall to obtain an in-plane image (Figure 1A).

The three muscle layers were infiltrated with BTA: external oblique, internal oblique, and transversus abdominis (Figure 1B).

### Injection sites

WE marked six sites, three on each side of the lateral abdominal wall (Figure 1C).

One must carefully choose the sites based on the ultrasound images, where the surgeon can visualize the three layers of muscle and achieve the most lateral position possible. The most cranial site is generally subcostal, at the level of the 9^th^ rib, and may be even more lateral, over the 10^th^ to 12^th^ ribs. The most caudal site is at or slightly above the iliac crest, and the middle site is the most lateral, at the average distance between the first two^9^.

### Dosage and dilution

There is a correct dose for each muscle group^13^, but currently there is no evidence on the ideal dose of BTA needed to achieve maximum relaxation of the abdominal muscles in pediatric patients. Based on other authors’ experiences^14^, we chose the highest dose reported as safe, 10 to 15U/kg.

Due to the limited space for infiltration, a volume of 1 to 2ml at each site was generally sufficient. Based on the patient’s weight, the total dose was calculated and diluted in a volume of 6 to 12ml and distributed equally among the six sites. Excessive volume can cause more pain and compromise ultrasound images.

### Closing time

Definitive closure was planned between 30 and 45 days after BTA application^9^.

During this period, dressings are applied with silver sulfadiazine^15^, sterile gauze, and bandages, with light compression at each change. Changes are more frequent in the first days due to membrane secretion, but they can be spaced out when scarification and membrane epithelialization begin.

### Definitive closure of the abdominal wall

Under general anesthesia and use of prophylactic cefazolin, the patient was placed in a supine position. Foley and nasogastric catheters were placed, two peripheral venous accesses, a central venous access can be used if necessary.

A median incision below the defect was made to enter the abdominal cavity. By opening and protecting the internal organs, the remaining sac was separated from the skin around its entire circumference, and the adhesions carefully divided.

The median incision was then extended cranially to the xiphoid process, and the edges of the defect were delimited and exposed. We actively searched for malrotation and performed the Ladd procedure as needed.

**Figure 2:**
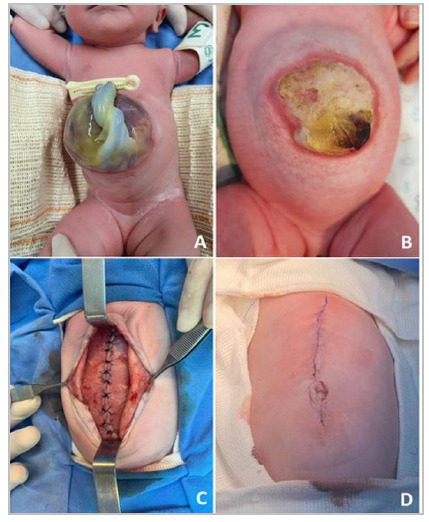
Photographic documentation of case 1. (A) GO at birth, measuring 11cm in its maximum diameter and an abdominal defect of 8.5cm in its lateral aspect. (B) Aspect of the defect on the day of surgical correction, 32 days after BTA application. (C) Closed midline without tension and without the need for additional techniques or the use of a prosthesis. (D) Immediate aesthetic result.

The adipocutaneous flaps were dissected laterally until there was enough space and the “corset” appearance came undone and the midline was easily approximated with tweezers (Figure 2C). At this time, monitoring the patient’s hemodynamics, intra-abdominal pressure (IAP), and ventilation patterns are crucial to ensure safe abdominal wall closure. If there are signs of compromise, other techniques for repairing the defect should be considered.

We sutured the aponeurosis with interrupted PDS 2-0 stitches, and fixed the skin to the aponeurosis with Vicryl 5-0. The skin was closed with an intradermal, absorbable suture.

A drain may be placed if there is excessive skin dissection.

Patients returned to the NICU, receiving prophylactic antibiotics for 24 hours. The decision to extubate was based on ventilatory needs and clinical judgment. No sedation or muscle paralysis is required if the IAP is at adequate levels and there have been no large increases in peak ventilatory pressure during closure. Oral feeding was started immediately after extubation.

## RESULTS

### Description of case 1

Female patient, born at our institution at 38 weeks of gestational age, weighing 2,530g, with a prenatal diagnosis of giant omphalocele. The abdominal wall defect measured about 8.5cm in its lateral extension, and the maximum diameter of the omphalocele was 11cm (Figure 2A). The sac contained more than 50% of the liver, stomach, and small intestine. A nasogastric catheter was placed, and an initial sterile dressing with gauze and bandages was performed. The echocardiogram showed dextrocardia, inter-atrial communication of the septum secundum of 4.6mm, with shunt from left to right and a patent ductus arteriosus of 2.3mm, with shunt from left to right. Abdominal ultrasound and karyotype were normal. Enteral feeding was started on the 3^rd^ day of life. BTA (Botox^®^) was applied on the 4th day of life, in a total dose of 12U/kg of BTA. Dressings with silver sulfadiazine, sterile gauze, and bandages were applied with light compression and were changed more frequently in the first week (every 2-3 days) and once every 5-7 days in the last week before surgical treatment. During the period between the application of BTA and the surgical correction, the patient remained in room air, with full enteral nutrition and breastfeeding, without the need for parenteral nutrition (PN) or antibiotics. Surgical correction was performed 32 days after BTA application (Figure 2B). The procedure was uneventful and primary closure was possible without the need for additional techniques or the use of a mesh (Figure 2C), with a final IAP of 6mmHg and an excellent immediate result (Figure 2D). Extubation occurred on the first postoperative day and discharge after nine days due to a slight oral aversion, with a total hospital stay of 43 days.

### Description of case 2

**Figure 3:**
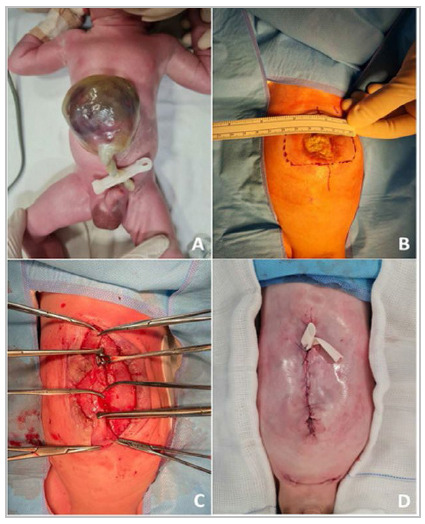
Photographic documentation of case 2. (A) GO at birth, measuring 12cm in its maximum diameter and an abdominal defect of 8cm in its lateral aspect. (B) GO on the day of surgical correction, 37 days after BTA application, with a 6cm defect. (C) Midline approach without tension. (D) Immediate aesthetic result.

Male patient born at 35 weeks and two days of gestational age weighing 2.100kg. The abdominal wall defect measured 8cm in its lateral extension, and the maximum diameter of the omphalocele was 12cm (Figure 3A), containing more than 50% of the liver, stomach, and small intestine. The echocardiogram showed a persistent ductus arteriosus and interatrial septal aneurysm, and the abdominal ultrasound showed mild bilateral dilatation of the renal pelvis. Bilateral inguinal hernias were observed at birth. No other major malformations were observed and the karyotype was normal. He received 14U/kg of BTA (Botox^®^) on the 2nd day of life. Dressings were done in the same way as in the other patient and full enteral feeding was achieved on the 7^th^ day of life. While awaiting surgical correction, the patient remained in room air, predominantly breastfed, without the need for PN, antibiotics, or a venous catheter, and in contact with his parents. He underwent the surgical procedure 37 days after the BTA application (Figure 3B). Primary closure was possible without the use of additional techniques or prostheses (Figure 3C), and a laminar drain was positioned subcutaneously (Figure 3D). The inguinal hernias were corrected during the same surgical procedure. The patient was extubated in the immediate postoperative period and oral feeding started. The drains were removed and the patient was discharged on the 5^th^ postoperative day. The total length of stay was 45 days.

### Postoperative follow-up

**Figure 4:**
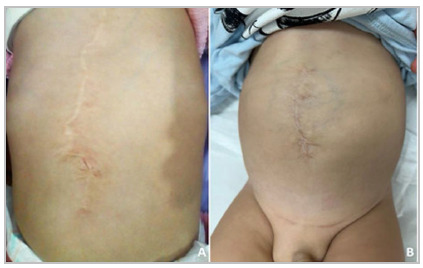
Long-term follow-up. (A) Case 1: 24-month follow-up result without signs of recurrence. (B) Case 2: 8-month follow-up result without signs of recurrence.

At the 24-month follow-up, the first patient had no complications and no sign of recurrence (Figure 4A).

The second child had two suture-related granulomas that drained spontaneously and did not require additional treatment. At the 8-month follow-up, the patient showed good child development, with no other complications or signs of recurrence (Figure 4B).

Both patients presented child development within normal standards, and the families demonstrated extreme satisfaction both during the treatment and with the surgical result.

## DISCUSSION

Despite decades of study and the description of various techniques, the treatment of GO is still the subject of debate. Currently, two main strategies are available: staged surgical closure and non-operative delayed closure, and there are data in the literature to support both approaches^7^ ^,^ ^8^.

Late, non-operative closure is usually performed with dressings and topical agents that stimulate scarification and epithelialization of the omphalocele membrane, and is followed by a late closure^8^ ^,^ ^15^. Key benefits include a shorter time to full enteral feeding and low morbidity.

Several techniques for staged surgical closure have been described to promote complete reduction of GO content, such as the placement of surgical silos^16^, non-surgical external silos^17^, intra-abdominal tissue expanders^18^, and external compression methods^19^. All these techniques are followed by an additional surgical intervention for the final closure. The main benefits of this approach include a shorter wall closure time and hospital discharge with corrected defect.

Most of these techniques require more than one procedure under general anesthesia, and these patients usually require mechanical ventilation, sedation, and/or muscle paralysis throughout the process, known to have repercussions on neurodevelopment in the long term^5^.

In addition, all these methods, including the non-operative approach, usually require the application of additional techniques at the time of surgery, such as component separation, skin or fascia flaps, negative pressure therapy, and/or use of prostheses^8^ ^,^ ^17^ ^,^ ^20^ ^-^ ^23^. The addition of any of these techniques significantly increases surgical morbidity, postoperative complications and recurrence rates^19^ ^,^ ^24^.

The benefits and effectiveness of applying BTA for repairing large abdominal wall defects in adults have been previously reported^9^, and there is evidence of its use for other purposes in children and neonates^14^ ^,^ ^25^.

We recently reported the first cases in the literature of the application of BTA in children with hernias secondary to GO^4^ ^,^ ^10^. The positive results aroused our interest in the exclusive use of BTA, mainly in neonates.

In both cases reported herein, the approximation of the midline was easily obtained, even in the subxiphoidal region, where the defect is usually larger, there is less aponeurosis, and the rigidity of the rib cage can hamper closure.

Both patients needed minimal manipulation, requiring sedation only during BTA application. They were maintained on complete enteral nutrition since the first week of life, including breast milk, and in close contact with their parents.

We observed only minor complications, and the aesthetic result was favorable in both cases. We found no adverse reactions related to the use of BTA, since they are rare^14^. However, when they do occur, they are usually transient or mild, while serious adverse reactions are rare and depend on the applied muscle group, being associated with higher doses^14^ ^,^ ^26^ ^,^ ^27^. The most worrisome complication is respiratory distress, although in such events, patients are already in the NICU and can be actively monitored and quickly managed. Care must be taken when administering BTA in patients using certain medications that may potentiate the effects of the toxin, such as aminoglycosides or non-depolarizing muscle relaxants, or in cases where it may result in the worsening of any pre-existing condition^13^ ^,^ ^26^.

The time for surgical correction can vary. The onset of BTA action appears to be within 48 hours of application, but its peak may occur between one and four weeks^13^ ^,^ ^28^. According to studies in adults, most groups indicate surgical correction between 30 and 45 days after the BTA application^9^, and the surgery performed approximately one month after BTA injection seems to be the most effective for repairing ventral hernias^29^. Therefore, based on our experience, we chose to intervene approximately 30 days after application.

Although it was possible to easily approximate the midline without additional techniques such as separation of the abdominal components or the use of prostheses, we recommend having a biological mesh available in case primary closure is not possible or there is hemodynamic repercussions at the time of midline approximation.

Our technique represents a middle ground between staged and late closure techniques. It does not require early aggressive interventions and the patient is discharged with the defect repaired, with a high probability of primary closure and without the need for additional techniques or prostheses. Although there are other reports in the literature on the use of BTA in children with GO, all are patients with a secondary ventral defect at an older age^4^ ^,^ ^10^ ^-^ ^12^. Therefore, this is the first article to report this approach in neonates and the first detailed description of the technique in this population.

We recognize that the patients in our study did not have major cardiovascular malformations or significant pulmonary hypoplasia, which certainly contributed to the favorable results. However, this does not negate that this approach leads to a more gentle process until closure by not requiring aggressive and invasive interventions.

Application of BTA to the abdominal wall is a relatively simple technique that can be performed in any center, given the availability of BTA and ultrasound equipment. The use of ultrasound is mandatory for proper visualization of the needle and toxin infiltration, minimizing possible complications.

Due to the complexity of GO, there will always be room for developing new techniques or the improvement of the existing ones. The objective of this study was to describe our technique for approaching GO and to report the first experience in the literature with the use of BTA in neonates, presenting an additional tool for managing this complex condition. However, this technique is still very new and we encourage studies to confirm its benefits and long-term results.
